# Clinical Characteristics and Outcomes of Asian Coral Snake Bites in Thailand: A Retrospective Cohort Study

**DOI:** 10.3390/toxins18040177

**Published:** 2026-04-06

**Authors:** Phantakan Tansuwannarat, Satariya Trakulsrichai, Juthathip Pathumarak, Achara Tongpoo

**Affiliations:** 1 Chakri Naruebodindra Medical Institute, Faculty of Medicine Ramathibodi Hospital, Mahidol University, Bangkok 10540, Thailand; 2 Ramathibodi Poison Center, Faculty of Medicine Ramathibodi Hospital, Mahidol University, Bangkok 10400, Thailand; 3 Department of Emergency Medicine, Faculty of Medicine Ramathibodi Hospital, Mahidol University, Bangkok 10400, Thailand

**Keywords:** *Calliophis*, *Sinomicrurus*, snake envenomation, clinical characteristics, neurotoxicity, poison center

## Abstract

Asian coral snakes are distributed throughout Southeast Asia, including Thailand, but clinical data on their envenomation remain limited. Using a 10-year retrospective dataset from the Ramathibodi Poison Center, we investigated the epidemiology, clinical characteristics, management, and outcomes of Asian coral snake envenomation in Thailand. Patient demographics, clinical and laboratory data, treatments, and outcomes were analyzed descriptively. Fifty-two patients were included. *Sinomicrurus macclellandi* was the most frequently reported species. Most bites occurred during the rainy season and involved the lower extremities. Clinical manifestations were predominantly mild and localized. No cases of systemic neurotoxicity, bulbar weakness, respiratory compromise, or death were observed. Laboratory results were generally within normal limits. Two patients developed anaphylaxis, which resolved with standard emergency treatment, while two experienced severe pain. *Calliophis intestinalis lineata* was associated with a higher proportion of tachycardia at presentation and longer hospitalization. No patients required mechanical ventilation or antivenom therapy. Supportive care and short-term hospital observation are generally sufficient in confirmed cases. The median duration of hospitalization was 1–3 day. Local manifestations were the predominant clinical findings following Asian coral snake envenomation in Thailand, and systemic neurotoxicity was not observed. These findings differ from reports of *Micrurus* envenomation, which primarily involve New World coral snakes, whereas the species implicated in Thailand belong to Old World genera.

## 1. Introduction

Snakebite envenomation remains a major global health concern associated with significant morbidity and mortality [1,2]. The burden is particularly notable in Southeast Asia, where rich biodiversity and agricultural activities increase human exposure to venomous snakes. In Thailand, snakebite is a frequently encountered medical emergency, and several elapid species account for most clinically significant envenomations. These include monocled cobra (*Naja kaouthia*), king cobra (*Ophiophagus hannah*), banded krait (*Bungarus fasciatus*), and Malayan krait (*Bungarus candidus*), which are well recognized for their neurotoxic effects and potential to cause respiratory failure [1,2,3,4,5]. Within the family Elapidae, coral snakes represent a distinct lineage characterized by small, fixed proteroglyphous fangs and typically vivid aposematic coloration [6].

Nearly 30 recognized species of Asian (Old World) coral snakes have been described and are currently classified into three principal genera: *Calliophis*, *Sinomicrurus*, and *Hemibungarus*. Although these snakes possess potent venoms, reports of human envenomation are relatively uncommon when compared with those for other elapids [6,7,8,9]. The low frequency of reported envenomation may reflect their reclusive behavior and the presence of fixed proteroglyphous fangs, which differ from those of other medically important elapids [6,10,11].

The composition of venom in Old World coral snakes differs substantially from that of their New World counterparts of the genus *Micrurus*. For example, proteomic analyses of the Malaysian banded coral snake (*Calliophis intestinalis*) have demonstrated that its venom is composed predominantly of phospholipases A_2_ (PLA_2_) and three-finger toxins (3FTx), with cytotoxins and cardiotoxins constituting a substantial proportion and neurotoxins being less abundant [6]. While many *Micrurus* species have venoms that are typically dominated by neurotoxic 3FTx and PLA_2_ components. Such findings suggest divergent evolutionary pathways and functional specialization between Old and New World coral snake venoms. Phylogenetic analyses indicate that these lineages diverged millions of years ago, and differences in ecological niches and prey preferences may have driven the evolution of distinct venom phenotypes and clinical effects [12,13,14]. The long-glanded blue coral snake (*Calliophis bivirgatus*) produces calliotoxin (δ-elapitoxin-Cb1a), a three-finger toxin that acts as a voltage-gated sodium channel activator rather than a postsynaptic nicotinic acetylcholine receptor antagonist [11]. This toxin shifts the activation threshold of NaV1.4 channels toward more hyperpolarized potentials and inhibits inactivation, resulting in sustained depolarization and spastic paralysis in prey. In contrast, neurotoxins identified in *Sinomicrurus* species act primarily at the neuromuscular junction as antagonists of nicotinic acetylcholine receptors on the postsynaptic membrane [15]. These mechanistic differences further highlight the heterogeneity of venom phenotypes within Asian coral snakes.

In the Americas, envenomation by *Micrurus* species is well documented and frequently associated with systemic neurotoxicity [7,16]. Reports suggest that approximately 30% of total significant cases develop moderate to major clinical outcomes, including neurological manifestations, such as muscle weakness, and respiratory compromise requiring ventilatory support [7]. In contrast, published reports on bites of coral snakes from Asia describe predominantly local manifestations, and systemic neurotoxicity appears to be uncommon [9]. Fatal outcomes attributable to Asian coral snake envenomation have rarely been documented.

Various species of Asian coral snakes are distributed in Thailand, particularly within the genera *Calliophis* and *Sinomicrurus* [10,17]. However, envenomation by these species is infrequent, and comprehensive clinical data remain limited. Most available information consists of isolated case reports or small case series, and the true clinical spectrum of toxicity in Thai patients has not been clearly defined.

To help bridge this research gap, the present study focused on confirmed envenomations involving species within the genera *Calliophis* and *Sinomicrurus*, including *Sinomicrurus macclellandi* (formerly classified under *Calliophis*). Given the limited existing data and the apparent discrepancy between clinical patterns of coral snake bite victims reported in Asia and those described in the Americas, further characterization of these envenomations is warranted. Therefore, this study sought to evaluate the clinical features, management strategies, and the resulting outcomes of Asian coral snake envenomation in Thailand.

## 2. Results

**Figure 1:**
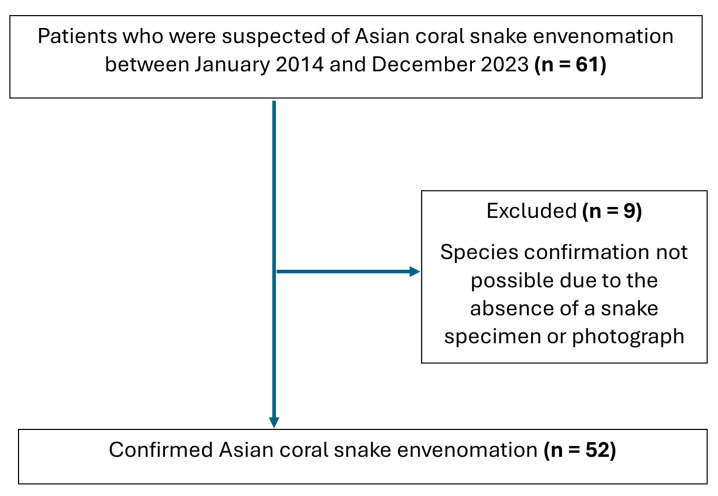
Flow diagram of Asian coral snake envenomation cases included in the study.

In total, 61 patients were initially considered to have suspected Asian coral snake envenomation based on a history of snakebite with a reported or presumed coral snake. Cases were included in this study when the biting snake was identified to the species level through direct examination of the specimen or expert review of photographs by a veterinarian specializing in snake identification at the Queen Saovabha Memorial Institute Snake Farm, with 52 cases meeting these criteria (Figure 1). Fourteen patients had clinical evidence of envenomation, and 38 cases were consistent with dry bite.

Table 1 summarizes the baseline demographic characteristics of the study population, which comprised 52 patients. *Sinomicrurus macclellandi* accounted for the largest proportion of cases (*N* = 30, 57.69%), followed by *Calliophis maculiceps* (*N* = 8, 15.38%), *Calliophis bivirgata flaviceps* (*N* = 7, 13.46%), and *Calliophis intestinalis lineata* (*N* = 7, 13.46%). The median (IQR) age of envenomed patients was 22 (11–41) years for *Calliophis bivirgata flaviceps*, 30 (5–37) years for *Calliophis intestinalis lineata*, 34.5 (17–42) years for *Sinomicrurus macclellandi*, and 43.5 (35–55.5) years for *Calliophis maculiceps.* Males represented the majority of patients in most species groups, although the sex distribution did not differ significantly between species (*p* = 0.436). Lower extremities were the most frequent bite sites across all species, accounting for 57.14–85.71% of cases. Patients bitten by *Sinomicrurus macclellandi* had the longest median time from bite to hospital presentation (median 1 h, IQR 0.50–2).

**Figure 2:**
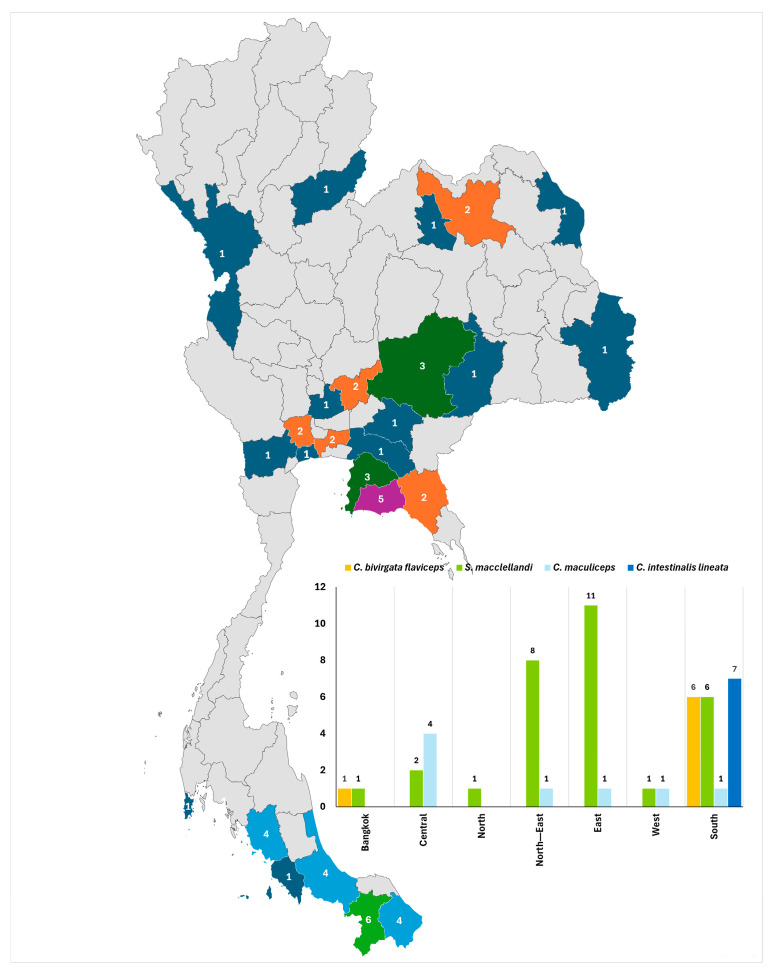
Geographic distribution of Asian coral snake envenomation cases by province and region in Thailand.

Cases were reported from multiple regions of Thailand, with distinct geographical patterns observed between species (Figure 2). For example, most bites from *Calliophis bivirgata flaviceps* (85.71%) and all bites from *Calliophis intestinalis lineata* (100%) occurred in the Southern region. In contrast, *Sinomicrurus macclellandi* bites were more widely distributed, occurring predominantly in the Eastern (36.67%) and Northeastern (26.67%) regions, with smaller proportions reported from the Southern and Central regions. *Calliophis maculiceps* bites were most frequently reported from the Central region (50.00%).

**Figure 3:**
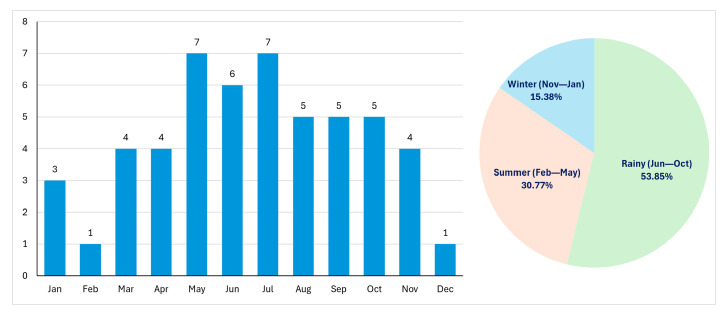
Monthly and seasonal distribution of Asian coral snake envenomation cases in Thailand.

Seasonal variation in Asian coral snake envenomation is shown in Figure 3. More than half of the cases occurred during the rainy season (53.85%), while 30.8% occurred in summer and 15.38% in winter. Case numbers increased from March onward, with peaks in May and July (7 cases each). The lowest frequencies were observed in February and December (1 case each).

Most cases were recorded between May and October. Clinical findings are shown in Table 2. The majority of patients experienced only mild localized symptoms. Fang marks were identified in the majority of patients with Asian coral snake envenomation in Thailand (66.67–100%). Local swelling was observed most often for *Calliophis bivirgata flaviceps* (57.14%) and less frequently for the other species. A minority of patients reported pain at the bite site, which was typically mild, although two patients experienced severe pain. Vital signs at presentation were largely within normal limits. Tachycardia differed significantly among species (*p* = 0.048) and was more frequently documented in patients bitten by *Calliophis intestinalis lineata*. Blood pressure at presentation did not differ significantly between species (*p* = 0.721). No patients developed systemic neurotoxicity. Specifically, no cases of ptosis, bulbar weakness, respiratory compromise, or paralysis were observed. Two cases of anaphylaxis were identified, one following envenomation by *Calliophis bivirgata flaviceps* and the other by *Sinomicrurus macclellandi*. Both patients received standard emergency management, including intramuscular adrenaline, dexamethasone, and chlorpheniramine. Clinical symptoms resolved after treatment, and no cases of refractory or biphasic anaphylaxis were observed.

Management was primarily supportive. Most patients were observed without specific intervention. Intravenous fluids were administered in a small proportion of cases. Antibiotics were prescribed in 23–43% of patients, depending on the species. No patients required mechanical ventilation, and no antivenom was administered. Most patients suffered no or only mild clinical effects. Local effects were reported in some cases, particularly pain in *Calliophis intestinalis lineata*. No fatalities occurred. Severe pain at presentation was documented in two patients, one bitten by *Calliophis bivirgata flaviceps* and one by *Sinomicrurus macclellandi*. Both patients required intravenous analgesics, such as tramadol, upon arrival at the hospital. The length of hospital stay differed among species . Patients bitten by *Calliophis intestinalis lineata* were admitted for longer than those bitten by other species. Significant differences were observed among species in the presence of tachycardia at presentation.

The number of patients tested for each parameter is shown in Table 3. Among those evaluated, laboratory findings did not reveal clinically significant abnormalities, with electrolyte and renal function parameters generally remaining within normal limits across species groups. No cases of acute kidney injury or clinically significant metabolic disturbances were identified among patients with available data.

## 3. Discussion

This study represents one of the largest clinical series of Asian coral snake envenomation reported from Thailand. Over a 10-year period, only 52 confirmed cases were recorded, emphasizing the relatively low incidence of these envenomations compared with those by other elapids in the region, such as cobras and kraits [4,18]. Most cases occurred between May and October, corresponding to the rainy season, when increased rainfall and localized flooding may disrupt natural habitats and drive snakes to seek shelter in areas with greater human activity. Meanwhile, intensified agricultural activity and reduced ground visibility during wet conditions may further increase the likelihood of human–snake encounters [19,20]. The geographical distribution of cases in this study was concordant with known species ranges, with *Calliophis bivirgata flaviceps* and *Calliophis intestinalis lineata* occurring predominantly in Southern Thailand, and *Sinomicrurus macclellandi* and *Calliophis maculiceps* distributed more broadly nationwide [10], supporting the reliability of species identification.

Clinically, the effects of envenomation in our cohort were generally mild and localized. No systemic neurotoxicity or respiratory failure was observed. In contrast, envenomation by New World coral snakes (genus *Micrurus*) in the Americas has been associated with neurological manifestations in some patients, sometimes requiring ventilatory support [7,16]. The absence of neurotoxicity in our study likely reflects species-specific venom characteristics rather than under-recognition, as all confirmed cases involved the genera *Calliophis* and *Sinomicrurus*.

Transcriptomic analyses of *Calliophis bivirgata flaviceps* venom gland demonstrated a predominance of 3FTx, mainly delta-neurotoxins, which are the most diverse and abundant components, showing significant sequence and functional variability compared to other Asiatic elapids [21]. This contrasts with many *Micrurus* species whose venoms are enriched in neurotoxic 3FTx, and PLA_2_ that interfere directly with neuromuscular transmission [12,13,14]. The divergence in venom characteristics between Old World and New World coral snakes may be related to ecological differences. Many *Micrurus* species are known to be ophiophagous, which may be associated with the need for rapid neuromuscular immobilization of prey and the evolution of potent α-neurotoxins. These differences in venom composition may partially explain the distinct clinical manifestations observed between Old and New World coral snakes. In contrast, available ecological data on *Calliophis* species remain limited. Although some species are considered ophiophagous, variation across species cannot be excluded, and their prey preferences remain incompletely characterized. These ecological differences and uncertainties may contribute to variation in toxin composition and may partly account for differences in clinical syndromes observed between regions [22,23,24]. However, these interpretations remain hypothesis-generating, as no venom analysis was performed in the present study, and should therefore be interpreted with caution.

Species-level variation in patient outcome as also observed within the genus *Calliophis*. For example, patients bitten by *Calliophis intestinalis lineata* demonstrated the highest proportion of clinical pain, tachycardia at presentation and median hospital stay when compared to the other species. Although case numbers were limited, these findings raise the possibility of interspecies differences in venom yield or toxin composition . The higher rate of tachycardia in this group may reflect sympathetic activation related to pain or stress rather than direct cardiotoxicity [25]. Antibiotics were used in 23–43% of patients, mainly in the presence of local wound findings when infection was suspected or could not be excluded. Current WHO snakebite management guidelines do not recommend routine prophylactic antibiotics; instead, their use should be limited to patients with clinical evidence of wound infection or a high risk of secondary contamination [26]. The relatively frequent use observed in this cohort may reflect real-world clinical practice and diagnostic uncertainty in early presentations. The longer hospitalization may similarly reflect caution by the physician rather than objective clinical deterioration. Larger studies are thus required to confirm these observations. From a clinical perspective, the immediate risk of respiratory failure following confirmed *Calliophis* and *Sinomicrurus* envenomation in Thailand appears to be low. None of the patients required ventilatory support or antivenom therapy, and most were discharged within 24 h. These findings suggest that short-term observation may be sufficient in the absence of evolving neurological signs. Nevertheless, monitoring during the first 24 h remains prudent given the neurotoxic potential described in other coral snake species [9,27].

Certain limitations should be taken into account when interpreting these findings. Foremost among these is the retrospective nature of the study and reliance on poison center consultation records, which introduces the possibility of incomplete or missing clinical data. The reporting of coral snake bites in Thailand to our poison center is not mandatory; therefore, the true national incidence of such cases may be underestimated. Second, the data were derived from a single national poison center in Thailand. Although this center provides nationwide consultation services, the findings may not be fully generalizable to other countries where differences in snake species distribution, healthcare access, and management protocols may influence clinical outcomes. Third, species identification was based on expert review of snake carcasses or photographs when available, without definitive laboratory confirmation (e.g., detection of venom components in biological samples). This may introduce misclassification bias, which could affect the accuracy of species-specific analyses. However, this approach reflects real-world clinical practice in many resource-limited settings. Therefore, the findings should be interpreted with caution. Fourth, certain laboratory investigations were not routinely performed. In particular, creatine kinase levels were not systematically measured; therefore, subclinical myotoxicity could not be completely ruled out. Nevertheless, no patients exhibited clinical features suggestive of rhabdomyolysis, such as myalgia or dark urine, and no clinically significant muscle injury was documented. Given the mild clinical course observed overall, this limitation is unlikely to have altered patient management. Finally, the relatively small number of cases, particularly within individual species groups, limits the statistical power to detect subtle interspecies differences. Observed variations in clinical severity and length of hospital stay should therefore be interpreted with caution and confirmed in larger multicenter studies.

## 4. Conclusions

This study represents one of the largest clinical series of confirmed Asian coral snake bites in Thailand. In this cohort, such bites were uncommon and were generally associated with mild, self-limited local manifestations. No systemic neurotoxicity or respiratory failure was observed, and no fatalities occurred. Although some species-level differences in presentation were noted, most patients required only supportive care. Because this was a retrospective study with a limited sample size and non-uniform clinical assessment, subtle or delayed neurological manifestations cannot be completely excluded. Therefore, careful monitoring during the first 24 h after a confirmed bite remains prudent.

## 5. Materials and Methods

This study utilized data from the Ramathibodi Poison Center, a division of Ramathibodi Hospital, a tertiary university hospital in Thailand that provides nationwide 24 h telephone consultation services for both medical personnel and the general population. All cases are recorded in the Ramathibodi Poison Center database. All records undergo thorough review and verification by experienced poison information specialists and clinical toxicologists. This analysis included all cases of Asian coral snake bite referred to our poison center for telephone consultation over the 10-year period from 2014 to 2023. Cases were ascertained by querying the database using the “substance” variable, with “Asian coral snake bite” being recorded as the substance of exposure. Asian coral snake bites were identified based on a history of snakebite with a reported or presumed coral snake and supported by one or more of the following: identification of the snake or carcass brought to the hospital by the patient or a witness, or photographic evidence provided by the patient or a witness. All available snake specimens or photographs were subsequently verified by a poison center consultant and an experienced veterinarian from the Snake Farm at Queen Saovabha Memorial Institute, Thai Red Cross Society, Bangkok, Thailand. Cases were classified as confirmed when the biting snake was identified to the species level through direct examination of the specimen or expert review of photographs. Species identification was further categorized based on the level of diagnostic confidence as definite (identification confirmed by direct examination of the snake specimen) or probable (identification based on expert review of photographic evidence). Both definite and probable cases were included in the final analysis. Cases without available specimens or photographs for expert verification were classified as suspected and excluded from the final analysis. Patients with co-exposures to pesticides, herbal products, illicit drugs, other chemicals, or medication overdoses that could influence clinical or laboratory findings were excluded. Cases with incomplete records were also excluded.

Envenomation was defined as the presence of local and/or systemic clinical effects attributable to snake venom. Cases with no clinical evidence of envenomation were included. A “dry bite” was defined as a bite by a venomous snake in which no venom is injected, resulting in the absence of envenomation despite the presence of fang marks. The presence of local symptoms (e.g., pain or swelling) indicates venom injection and therefore does not meet the definition of a dry bite. In cases with incomplete or ambiguous clinical information, classification was determined by consensus among experienced poison center consultants and clinical toxicologists based on all available clinical data. The dataset included patient demographics, medical history, laboratory findings, treatments administered, follow-up information, final diagnoses, and outcomes. Laboratory investigations at initial presentation were performed at the discretion of the treating physicians and were not standardized across all cases.

Descriptive analyses were performed after data entry into a Microsoft Excel spreadsheet (Microsoft Corp., Redmond, WA, USA), which was also used for data visualization. Statistical analyses were conducted using Stata version 18 (StataCorp, College Station, TX, USA). Continuous variables are presented as mean ± standard deviation for normally distributed data and as median (interquartile range, IQR) for non-normally distributed data. Categorical variables are summarized as frequencies and percentages. Comparisons of normally distributed continuous variables were performed using one-way analysis of variance, whereas the Kruskal–Wallis test was applied for non-normally distributed data. Differences in categorical variables were assessed using the chi-square test or Fisher’s exact test, as appropriate. A *p*-value <0.05 was considered statistically significant.

## Figures and Tables

**Table 1:** Baseline characteristics and clinical presentations of Asian coral snake envenomation.

Variable	*Calliophis bivirgata flaviceps* (n = 7)	*Calliophis* *intestinalis lineata* (n = 7)	*Sinomicrurus* *macclellandi* (n = 30)	*Calliophis* *maculiceps* (n = 8)	*p* Value
Sex, N (%), Male	5 (71.43)	3 (42.86)	21 (70.00)	4 (50.00)	0.436
Age (years), median (IQR)	22 (11–41)	30 (5–37)	34.5 (17–42)	43.5 (35–55.50)	0.1591
Bite site, N (%) upper extremity lower extremity	3 (42.86) 4 (57.14)	1 (14.29) 6 (85.71)	10 (33.33) 20 (66.67)	2 (25.00) 6 (75.00)	0.686
Time to hospital from bite (hours), median (IQR)	0.50 (0.5–2)	0.50 (0.33–1)	1 (0.50–2)	0.75 (0.5–2.5)	0.1647

IQR: interquartile range.

**Table 2:** Clinical manifestations and management of patients with Asian coral snake envenomation.

Variables	*Calliophis* *bivirgata flaviceps* (n = 7)	*Calliophis* *intestinalis* *lineata* (n = 7)	*Sinomicrurus macclellandi* (n = 30)	*Calliophis* *maculiceps* (n = 8)	*p* Value
Pulse rate, N (%)					
Tachycardia	3 (42.86)	4 (57.14)	5 (16.67)	4 (50.00)	0.048
Blood pressure, N (%)					
Hypertension	3 (42.86)	2 (28.58)	6 (20.00)	6 (75.00)	0.721
Local effects, N (%)					
Observed fang mark	6 (85.71)	6 (85.71)	20 (66.67)	8 (100.00)	0.111
Wound swelling	4 (57.14)	2 (28.57)	6 (20.00)	1 (12.50)	0.1777
Pain at the bite site (%)	2 (28.57)	3 (42.86)	3 (10.00)	0	0.058
Management, N (%)					
Intravenous fluid	2 (28.57)	2 (28.57)	2 (6.67)	0	0.108
Antibiotic drug	2 (28.57)	3 (42.86)	7 (23.33)	2 (25.00)	0.781
Hospital stay (days), median (IQR)	2 (1–3)	3 (1–3)	1 (1–2)	2 (1–2.50)	0.0597

**Table 3:** Laboratory findings at presentation in patients with Asian coral snake envenomation.

Laboratory Parameter	*Calliophis* *bivirgata flaviceps* (n = 4)	*Calliophis* *intestinalis lineata* (n = 4)	*Sinomicrurus* *macclellandi* (n = 11)	*Calliophis* *maculiceps* (n = 0)
Serum sodium (mmol/L), mean ± SD	140 ± 2.08	138 ± 4.08	139 ± 3.55	NA
Serum potassium (mmol/L), mean ± SD	4.03 ± 0.49	3.9 ± 0.61	3.84 ± 0.41	NA
Serum chloride (mmol/L), mean ± SD	103 ± 2.49	107 ± 5.19	104 ± 3.79	NA
Serum bicarbonate (mmol/L), mean ± SD	25.7 ± 5.04	20.8 ± 5.91	24.7 ± 3.21	NA
Serum blood urea nitrogen (mg/dL), mean ± SD	13.4 ± 5.25	8.46 ± 3.4	11.4 ± 3.23	NA
Serum blood creatinine (mg/dL), mean ± SD	0.63 ± 0.17	0.63 ± 0.37	0.79 ± 0.25	NA

## Data Availability

The original contributions presented in this study are included in the article. Further inquiries can be directed to the corresponding author.
